# Exploring the causal connection: insights into diabetic nephropathy and gut microbiota from whole-genome sequencing databases

**DOI:** 10.1080/0886022X.2024.2385065

**Published:** 2024-08-01

**Authors:** Rui Lin, Rongping Chen

**Affiliations:** a The Second School of Clinical Medicine, Southern Medical University, Guangzhou, China; b Department of Endocrinology, Zhujiang Hospital, Southern Medical University/The Second School of Clinical Medicine, Southern Medical University, Guangzhou, China

**Keywords:** Gut microbiota, diabetic nephropathy, mendelian randomization, whole-genome sequencing

## Abstract

Over recent years, the prevalence of diabetes has been on the rise, paralleling improvements in living standards. Diabetic nephropathy (DN), a prevalent complication of diabetes, has also exhibited a growing incidence. While some clinical studies and reviews have hinted at a link between diabetic nephropathy and gut microbiota (GM), the nature of this connection, specifically its causative nature, remains uncertain. Investigating the causal relationship between diabetic nephropathy and gut microbiota holds the promise of aiding in disease screening and identifying novel biomarkers. In this study, we employed a two-sample Mendelian randomization analysis. Our dataset encompassed 4,111 DN patients from the GWAS database, juxtaposed with 308,539 members forming a control group. The aim was to pinpoint specific categories within the vast spectrum of the 211 known gut microbiota types that may have a direct causal relationship with diabetic nephropathy. Rigorous measures, including extensive heterogeneity and sensitivity analyses, were implemented to mitigate the influence of confounding variables on our experimental outcomes. Ultimately, our comprehensive analysis revealed 15 distinct categories of gut microbiota that exhibit a causal association with diabetic nephropathy. In summary, the phyla Bacteroidota and Verrucomicrobiae, the families Peptostreptococcaceae and Veillonellaceae, the genus Akkermansia, and the species Catenibacterium, Lachnoclostridium, Parasutterella, along with the orders Bacteroidales and Verrucomicrobiales, and the class Bacteroidetes were identified as correlates of increased risk for DN. Conversely, the family Victivallaceae, the species Eubacterium coprostanoligenes, and the Clostridium sensu stricto 1 group were found to be associated with a protective effect against the development of DN.These findings not only provide valuable insights but also open up novel avenues for clinical research, offering fresh directions for potential treatments.

## Introduction

Diabetic nephropathy (DN) ranks among the most prevalent microvascular complications of diabetes mellitus, bearing a substantial burden of morbidity and mortality in diabetic patients [1]. Over the past decade, China has experienced a dramatic escalation in the incidence and prevalence of diabetic nephropathy (DN), with approximately 24.3% of individuals with diabetes mellitus also suffering from chronic kidney disease (CKD), contributing to the global burden of DN, which affects an estimated 850 million people, predominantly due to the rising prevalence of DM. [2,3]. Several intricate pathways and mediators come into play in the initiation and progression of DN [4], including factors like oxidative stress, angiotensin II, and inflammatory processes, which are now acknowledged for their pivotal role [5]. The principal risk factors encompass hyperglycemia, hypertension, obesity, smoking, ethnicity, gender, dyslipidemia, age, and genetic predisposition, collectively influencing the development and advancement of DN [6,7].

Intriguingly, the gut microbiota, the body’s largest symbiotic microbial community, is an oft-overlooked player in this context. This intricate ecosystem comprises bacteria, fungi, viruses, and protozoa, tallying up to 4 trillion microorganisms and 150,000 microbial genomes [8]. Extensive research has posited specific patterns within the gut microbiome patterns are closely entwined with the onset of various chronic ailments in humans, encompassing nonalcoholic fatty liver disease, colorectal cancer, alcoholic hepatitis, and inflammatory bowel disease [9–13]. Recent investigations have hinted at a potential link between the gut microbiota and diabetic nephropathy. Notably, patients with Type 2 diabetes (T2D), particularly those afflicted with DN, exhibited significantly reduced viral richness and diversity compared to their healthy counterparts. A range of viral functions, particularly those executed by phages targeting host bacteria, exhibited notable depletion in T2D and DN [14]. Nonetheless, whether a causal nexus binds the gut microbiota to DN remains an enigma.

Mendelian randomization (MR) has emerged as a statistical method hinging on whole-genome sequencing data that effectively mitigates bias and elucidates causative relationships [15]. MR offers a means to scrutinize the presence of a causal link between an exposure and a specific outcome. In this study, we employ a two-sample MR analysis to delve into the potential causal association between gut microbiota and DN. Our exploration hinges on summary statistics from genome-wide association studies (GWASs), generously provided by the MiBioGen and FinnGen consortia.

## Materials and methods

### Study design

**Figure 1.**
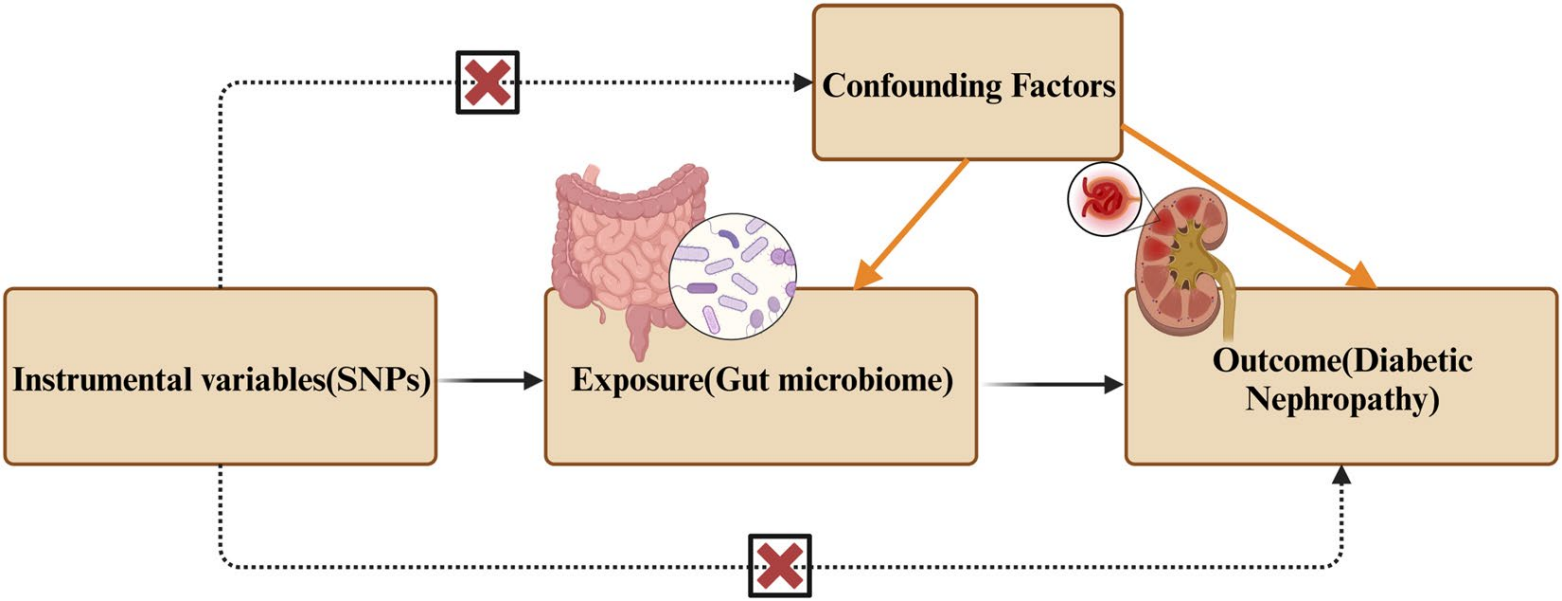
The three primary assumptions of MR.

Our research aimed to explore the potential link between gut microbiota and diabetic nephropathy through a two-sample Mendelian randomization approach. We leveraged data from GWAS databases, with gut microbiota information collected from MiBioGen and diabetic nephropathy data from FinnGen. To enhance the reliability of our findings and minimize the influence of confounding variables, we operated under the following three fundamental MR assumptions: (1) Selection of SNPs that exhibit significant associations with gut microbiota as instrumental variables (IVs). These IVs should demonstrate a robust correlation with the exposure, which is gut microbiota in this context. (2) Ensuring there is no inherent relationship between the IVs and the outcome variable, diabetic nephropathy. Any association observed should be solely mediated through the effect of the IVs on the exposure. (3) Confirming that the IVs have no connection to potential confounding factors (Figure 1).

### Ethics statement

Our research drew upon publicly available data from GWAS databases. Since these datasets are preexisting and publicly accessible, there was no need for ethical committee approval. It is important to note that each of the studies included in this article had already undergone review and received ethical clearance from their respective institutions or committees.

### Data sources

Summary-level data was collected from the MiBioGen databases, focusing on the human gut microbiota. This comprehensive dataset covered 211 bacterial taxa units, encompassing 131 genera, 35 families, 20 orders, 16 classes, and 9 phyla. Furthermore, we accessed summary statistics for DN from a dataset stored within the FinnGen biobank analysis round 11. This dataset contained information on 4,111 DN cases and 308,539 control subjects. By accessing the MiBioGen database official website and setting a P-value threshold (P = 1e-5), the target data was obtained and subsequently downloaded locally. Similarly, data for diabetic nephropathy was sourced from the FinnGen database. The vast majority of data in these databases originates from European samples, with a smaller portion derived from Asian, African, and admixed populations. All statistical analyses were performed using the R software(Version 4.3.1). The R package ‘TwoSampleMR’ was used to perform MR analysis of the causal relationship between gut microbiota and DN.

### Instrumental variables selection

Our first step involved the careful selection of instrumental variables strongly associated with the gut microbiota. To ensure a robust instrument selection, we employed a stringent statistical threshold, specifically a *p*-value threshold of <1 × 10^−5^, drawing upon established methodologies [16–18]. Furthermore, we implemented a threshold for the linkage disequilibrium metrics, R^2^ (set at 0.001), and KB (set at 10,000), with the intention of reducing the influence of SNP linkage. Our second step focused on aligning the exposure and outcome data by prioritizing SNPs that share the same alleles, thereby excluding SNPs with palindromic or incompatible characteristics.

In order to gauge the potential impact of weak instrument bias on our causal estimates, we assessed the strength of our instrumental variables. An instrumental variable was considered robust when its corresponding *F*-statistic surpassed a threshold of >10, indicating the absence of significant weak instrumental bias. Each SNP, the F statistic was calculated using the formula $F=\frac{R^{2}\times (N-2)}{1-R^{2}}$ where $R^{2}=\frac{2\times \beta^{2}\times \text{EAF}\times (1-\text{EAF})}{2\times \beta^{2}\times \text{EAF}\times (1-\text{EAF})+2\times {\text{SE}}^{2}\times N\times \text{EAF}\times (1-\text{EAF})}$, N represents the number of participants, EAF represents the effect allele frequency, and β is the estimated effect of the SNP to assess its ability to uniquely predict the outcome^1-3^.

### Mendelian Randomization analysis

**Figure 2.**
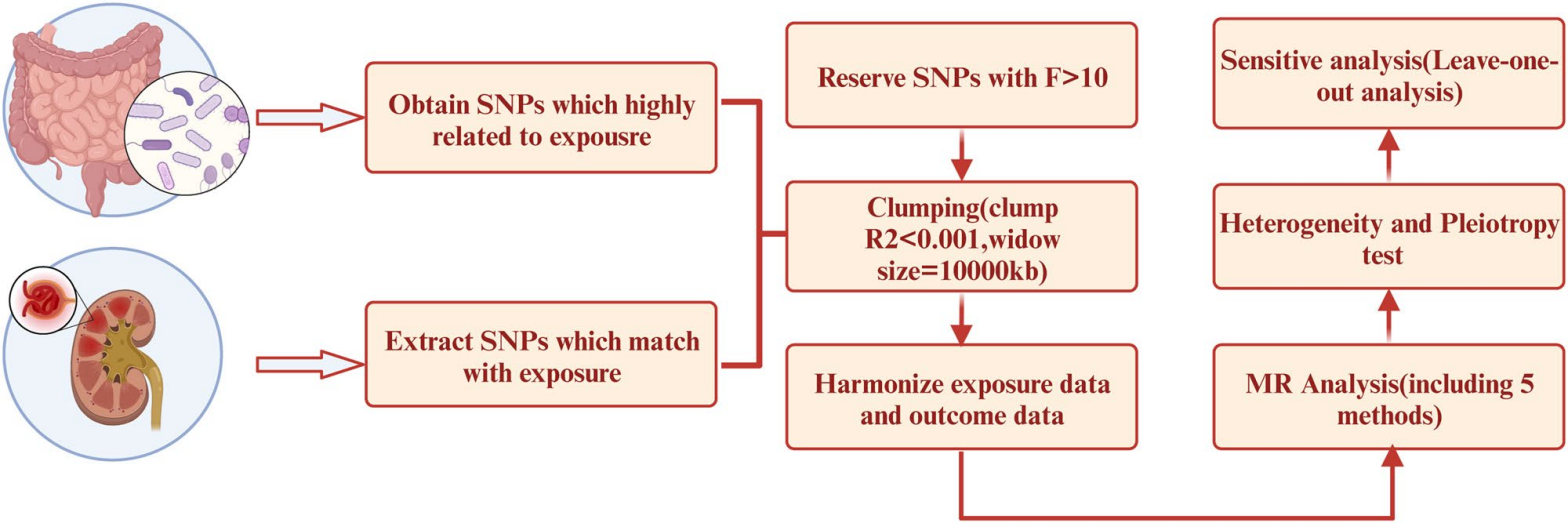
Flowchart outlining the design of Mendelian Randomization analysis.

Our MR analysis incorporated five fundamental methodologies, comprising MR Egger, Weighted Median (WM), Inverse Variance Weighted (IVW), Simple Mode, and Weighted Mode. IVW served as the primary approach, while the other methods were supplementary tools for corroboration. Following the data harmonization process, we initiated the MR analysis with the IVW method. If the resulting *p*-value was below 0.05, this initial step provided a preliminary indication of a potential causal relationship between the gut microbiota and DN. In cases where the *p*-value exceeded 0.05, this indicated a lack of statistical significance, and further validation became necessary, involving the application of MR Egger or alternative methods. Subsequently, a crucial heterogeneity test was performed, primarily evaluating the *p*-value derived from Cochran’s Q test. If this test yielded a *p*-value greater than 0.05, it signified the absence of heterogeneity. Conversely, the presence of heterogeneity necessitated the removal of biased SNPs using the MR-PRESSO tool. The subsequent stage involved conducting a pleiotropy test, with a focus on observing the *p*-value. If the *p*-value exceeded 0.05, the outcomes suggested the absence of statistical significance, indicating the absence of pleiotropy. Lastly, a sensitivity analysis was carried out to further evaluate and enhance the robustness of the findings (Figure 2). Moreover, the leave-one-out results further validated data robustness.

## Results

### SNP characteristics

Data regarding the exposure variables originate from the Mibiogen database. The gut microbiota exposure data encompass a compendium of 24 cohort studies conducted across as a spectrum of locations, including the United States, Canada, Israel, South Korea, Germany, Denmark, the Netherlands, Belgium, Sweden, Finland, and the United Kingdom. The dataset incorporates information on 211 distinct intestinal biological categories, spanning Actinobacteria, Bacteroides, Clostridia, and more. As for the outcome variables related to DN, they are drawn from the FinnGen database, encompassing 4,111 DN patients and 308,539 control individuals of European descent. The total number of SNP_S_ within this dataset reaches 18,708,278, following the elimination of instrumental variables in linkage disequilibrium. The final set of instrumental variables comprises 5 taxa and 15 unique bacterial characteristics, which comprises 2 classes, 4 families, 6 genera, 2 orders, and 1 phylum. Additionally, we have gathered a wealth of supplementary SNP information, including effect alleles, beta values, SE, and *p*-values, all featuring *F*-statistics exceeding the threshold of 10. For an exhaustive reference, please consult Supplementary Table S1.

### Causal influence of gut microbiota on DN

**Figure 3.**
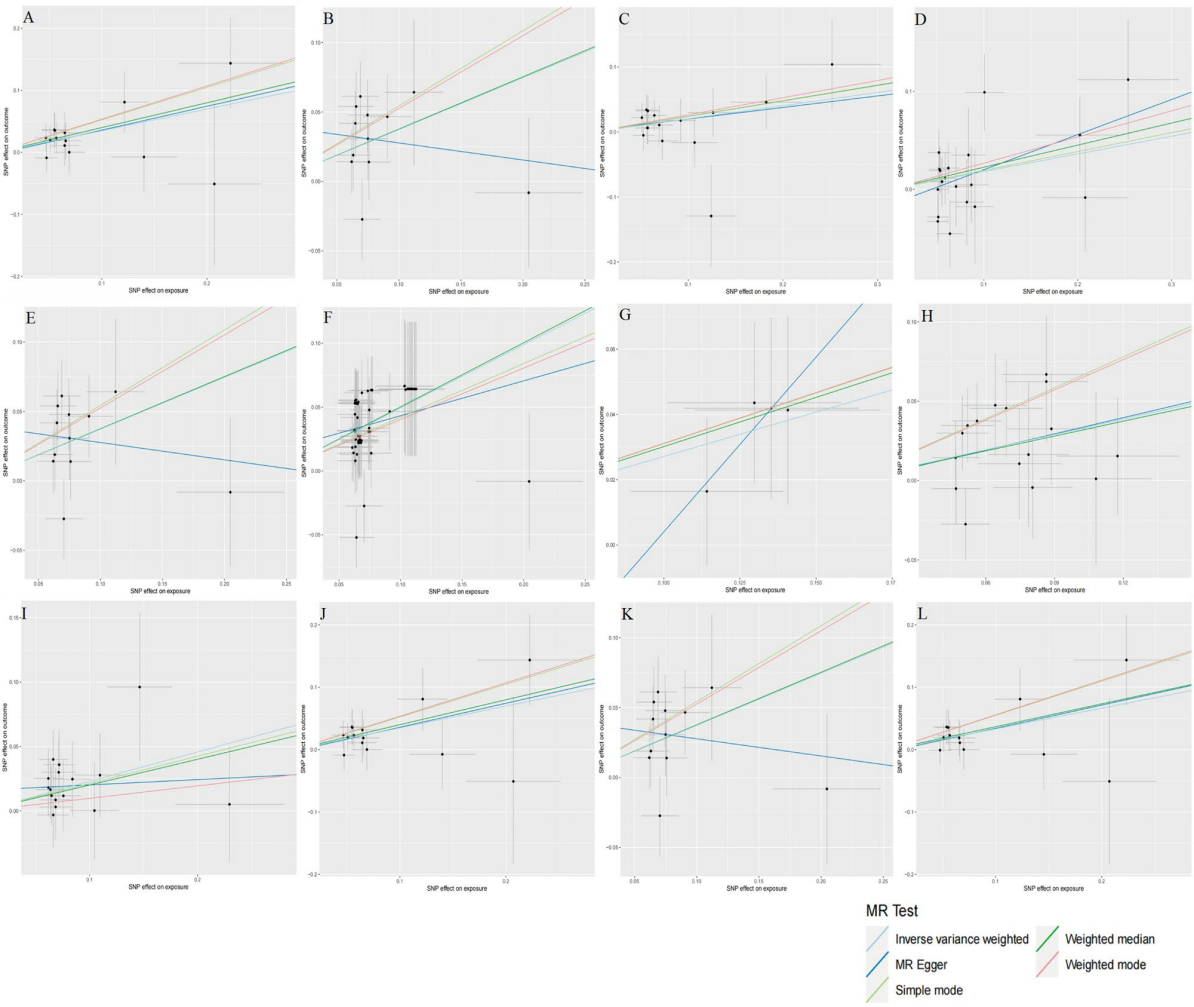
Summary of scatter plots depicting possible positive correlations between the gut microbiome and AD risk (a–L). Each data point on the graph represents a distinct SNP locus. The vertical axis signifies the influence of the instrumental variable on the outcome, while the horizontal axis represents the impact of the instrumental variable on the exposure. The ratio of these effects signifies the exposure’s influence on the outcome, effectively translating to the slope of the regression line reflecting the causal effect of exposure on the outcome in the graph. The horizontal and vertical crosses serve to illustrate the 95% confidence interval for each association. Although minor variations were observed in the estimates for the MR analysis, the overarching trend suggests a positive causal effect of the exposure (the gut microbiome) on the outcome (DN). Abbreviations: DN, diabetic nephropathy; SNPs, single nucleotide polymorphisms; MR, Mendelian randomization; IVW, inverse variance weighting.

**Figure 4.**
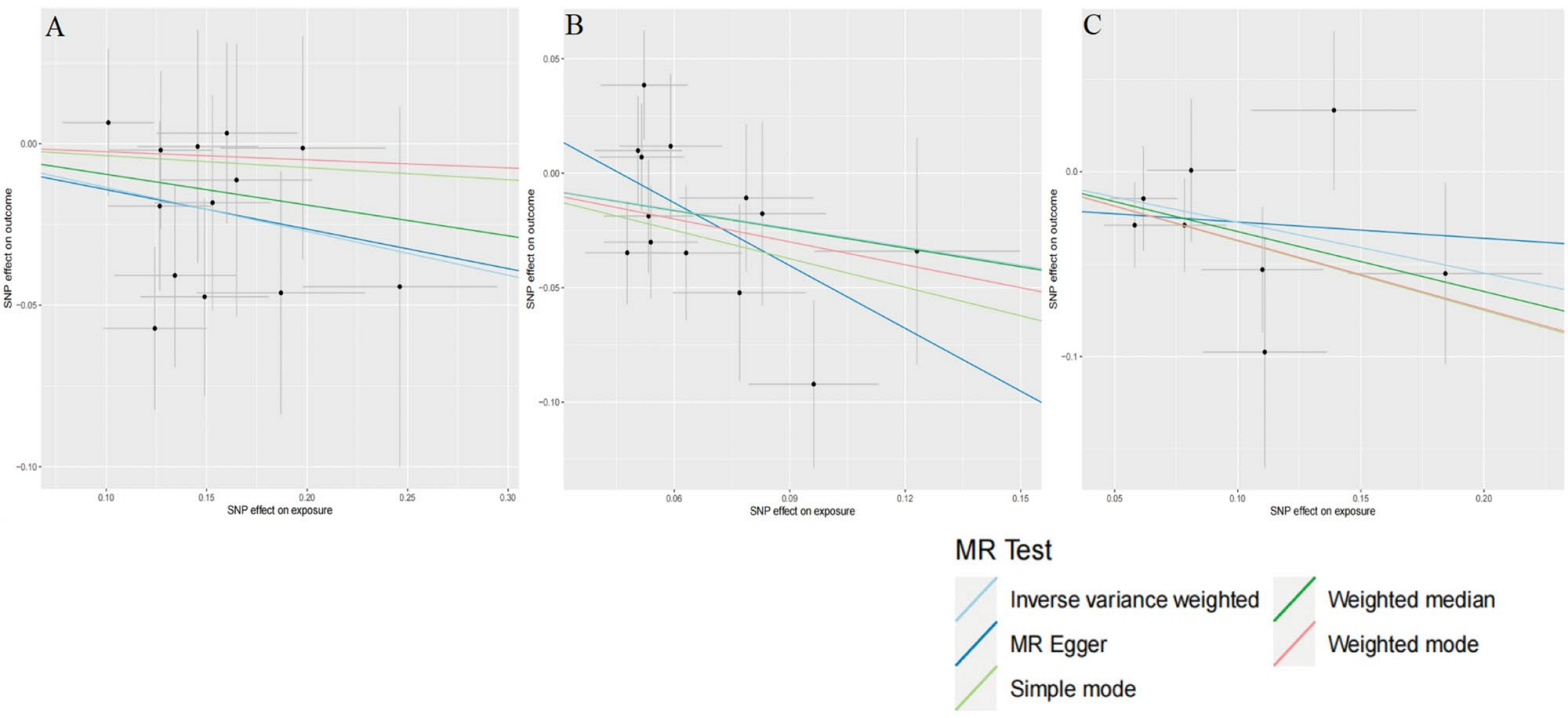
Depiction of the scatter plot summary illustrating the possible negative correlations between the gut microbiome and the risk of DN (a–C). Estimates derived from IVW estimates in these plots reveal that victivallaceae, the *Eubacterium coprostanoligenes* group, and *clostridium sensu stricto 1* exhibit a negatively sloping trend, implying a potential negative relationship between these microbial components and DN risk. Abbreviations: DN, diabetic nephropathy; SNPs, single nucleotide polymorphisms; MR, Mendelian randomization; IVW, inverse variance weighting.

**Figure 5.**
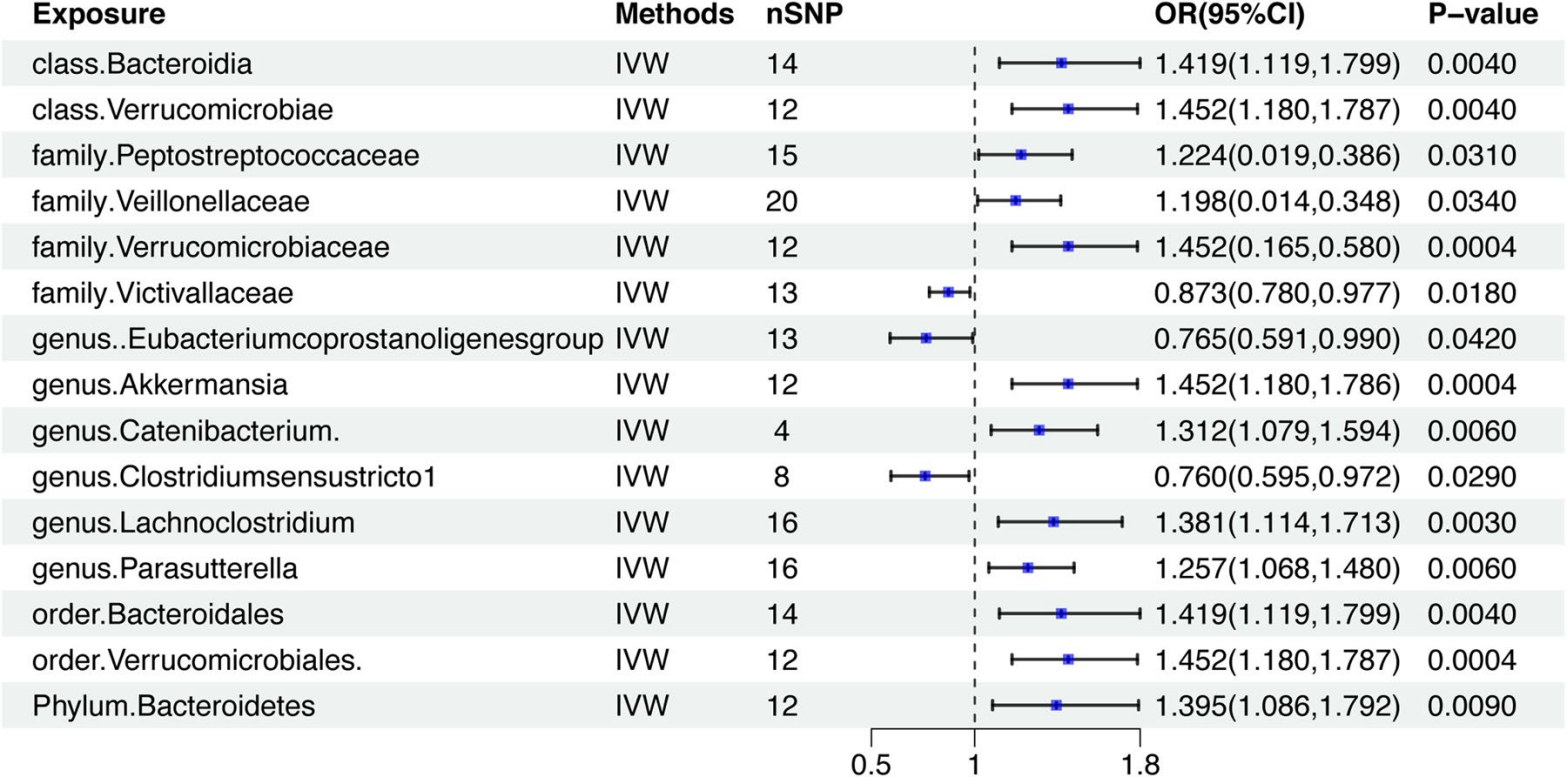
Forest Plot depicting the findings of IVW analysis on the composition of the gut microbiome and its impact on DN.

**Figure 6.**
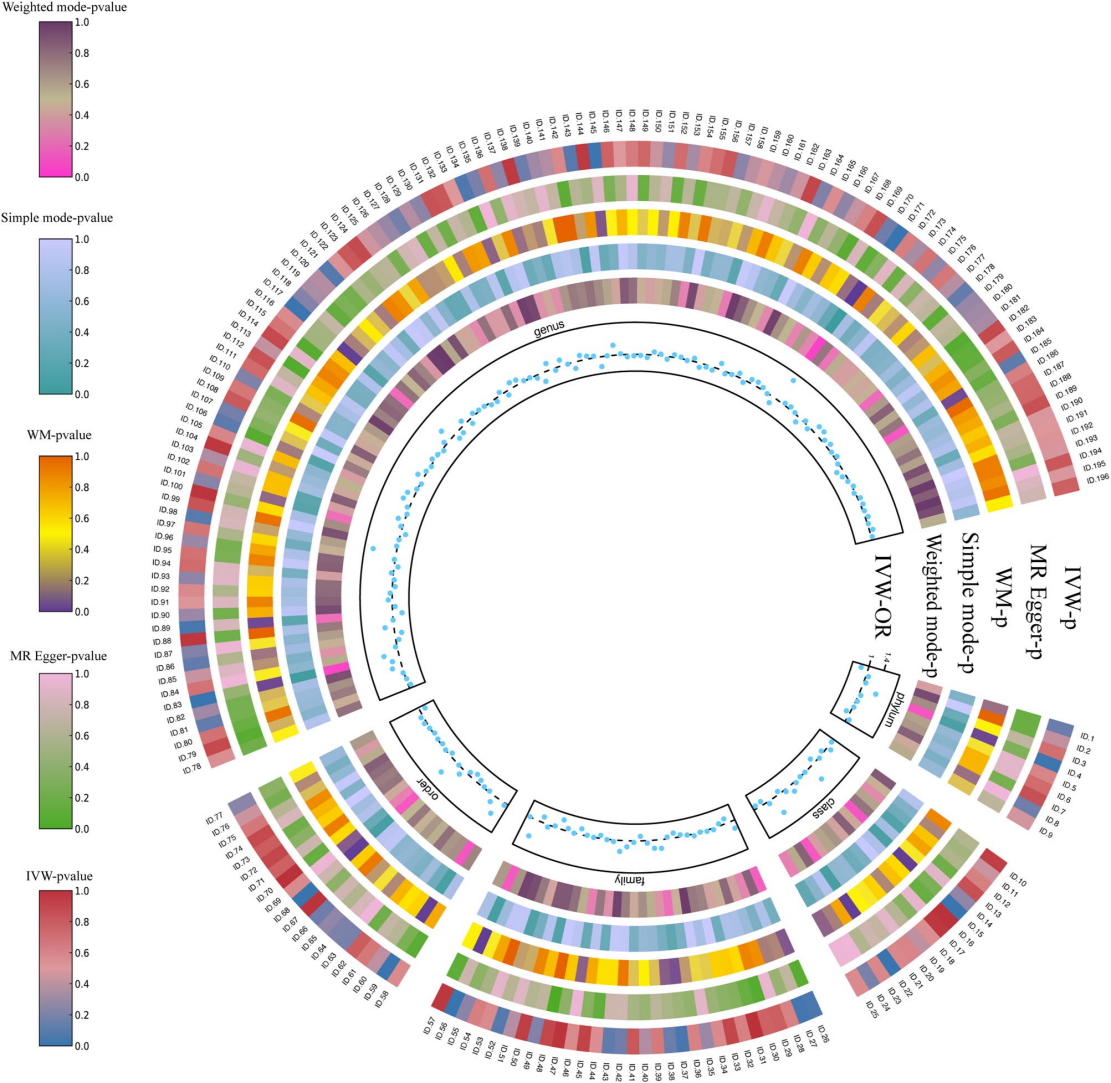
Causal examination of the gut microbiome taxa and their relationship with diabetic nephropathy using MR analyses. Focusing on loci with high significance (*p* < 1 × 10^−5^). In the concentric circles, the *p*-values for various MR analysis methods, including IVW, MR egger, WM, Simple mode, and Weighted mode, are presented, offering comprehensive insights into the causal associations. For the specific identification of the gut microbiome taxa represented by each ID, please refer to Supplementary Table S3.

Upon scrutinizing the causal effects at the phylum level, two gut microbiota elements surfaced as contributing positively to the development of DN. Specifically, Bacteroidota (OR = 1.419, CI = 1.119–1.799, *p* = 0.004), and Verrucomicrobiae (OR = 1.452, CI = 1.180–1.787, *p* = 0.004) were linked to an elevated risk of DN. Likewise, at the order level, Bacteroidales (OR = 1.419, CI = 1.119–1.799, *p* = 0.004), and Verrucomicrobiales (OR = 1.452, CI = 1.180–1.787, *p* = 0.0004) displayed a positive causal association with DN development. Switching our focus to the family level, it was observed that Victivallaceae (OR = 0.873, CI= 0.780–0.977, *p* = 0.018) exhibited a potential to mitigate the risk of DN, while Peptostreptococcaceae (OR = 1.224, CI = 1.019–1.471, *p* = 0.031), Veillonellaceae, (OR = 1.198, CI = 1.014–1.416, *p* = 0.034), and Verrucomicrobiaceae (OR = 1.452, CI = 1.180–1.787, *p* = 0.0004) displayed an inclination to increase the risk of DN. Upon closer examination, at the genus level, it emerged that *Akkermansia* (OR = 1.452, CI = 1.180–1.786, *p* = 0.0004), *Catenibacterium* (OR = 1.312, CI = 1.079–1.594, *p* = 0.006), *Lachnoclostridium* (OR = 1.381, CI = 1.114–1.713, *p* = 0.003), and *Parasutterella* (OR = 1.257, CI = 1.068–1.480, *p* = 0.006) were associated with an elevated risk of DN. Conversely, *Eubacterium coprostanoligenes* (OR = 0.765, CI = 0.591–0.990, *p* = 0.042), and *Clostridium sensu stricto 1* (OR = 0.760, CI = 0.595–0.972, *p* = 0.029) exhibited a potential to decrease the risk of DN. Zooming out to the phylum level, an abundance of Bacteroidetes (OR = 1.395, CI = 1.086–1.792, *p* = 0.009) signaled a significantly increased risk of DN. To sum up, Bacteroidota, Verrucomicrobiae, Peptostreptococcaceae, Veillonellaceae, Verrucomicrobiaceae, *Akkermansia*, *Cateni­bacterium*, *Lachnoclostridium*, *Parasutterella*, Bacteroidales, Verrucomicrobiales, and Bacteroidetes were identified as risk factors for DN, while Victivallaceae, *Eubacterium coprostanoligenes*, and *Clostridium sensu stricto 1* emerged as protective factors against DN. Complete scatter plots are presented in Figure 3 and Figure 4. The forest plot, based on IVW analysis results, is illustrated in Figure 5, while Figure 6 delves into the causal analysis of the gut microbiome taxa and DN, grounded in MR analyses.

### Rigorous sensitivity assessment

To ensure the robustness of our findings, we conducted an extensive sensitivity analysis. This comprehensive analysis encompassed assessments for both pleiotropy and heterogeneity. In the investigation of pleiotropy, we applied the MR-Egger method and MR-PRESSO analysis, and reassuringly, neither approach detected any potential horizontal pleiotropic effects, with all *p*-values exceeding 0.05. Turning to the assessment of heterogeneity, the examination of Cochran’s Q *p*-value, surpassing 0.05, coupled with the leave-one-out analysis, consistently pointed to the absence of heterogeneity. The comprehensive outcomes of this sensitivity analysis are detailed in Table 1, and more extensive information can be found in Supplementary Table S2.

**Table 1.** Sensitivity analysis results.

Classification	Horizontal pleiotropy			Heterogeneity		MR-PRESSO
	Egger	SE	*p-*value	Cochran’s	*p*-value	
	Intercept			Q methods		
Phylum: Bacteroidetes	−0.003	0.022	0.89	MR Egger	0.864	0.903
				IVW	0.91	
Order: Bacteroidales	−0.002	0.021	0.903	MR Egger	0.913	0.935
				IVW	0.944	
Order: Verrucomicrobiales	0.04	0.028	0.184	MR Egger	0.501	0.473
				IVW	0.413	
Class: Bacteroidia	−0.003	0.021	0.903	MR Egger	0.913	0.94
				IVW	0.944	
Class: Verrucomicrobiae	0.04	0.028	0.184	MR Egger	0.501	0.44
				IVW	0.413	
Family: Peptostreptococcaceae	0.002	0.172	0.901	MR Egger	0.746	0.829
				IVW	0.801	
Family: Veillonellaceae	−0.016	0.014	0.258	MR Egger	0.507	0.489
				IVW	0.483	
Family: Verrucomicrobiaceae	0.04	0.278	0.182	MR Egger	0.501	0.45
				IVW	0.412	
Family: Victivallaceae	−0.002	0.04	0.961	MR Egger	0.78	0.861
				IVW	0.842	
Genus: *Eubacterium coprostanoligenes*	0.042	0.031	0.2	MR Egger	0.41	0.35
				IVW	0.346	
Genus: *Akkermansia*	0.016	0.018	0.39	MR Egger	0.982	0.463
				IVW	0.982	
Genus: *Catenibacterium*	−0.103	0.16	0.59	MR Egger	0.923	0.913
				IVW	0.902	

## Discussion

We conducted a comprehensive two-sample MR analysis to uncover the potential causal links between gut microbiota and DN using public data from GWAS. Previous investigations primarily explored the relationship between these factors through clinical trials and animal models [19,20]. While these endeavors did establish a correlation between gut microbiota and DN, they were inherently vulnerable to confounding factors, rendering it challenging to conclude definitively whether a causal link exists. Our MR analysis now provides strong evidence indicating a causal association between specific gut microbiota and the risk of DN. Importantly, these findings remained robust in the face of potential sources of distortion such as heterogeneity or horizontal pleiotropy. This breakthrough discovery may serve as a stepping stone for the identification of novel biomarkers in future research on DN.

The gut microbiota represents a dynamic community of microorganisms, comprising a staggering 100 trillion microbes thriving within the host organism’s gastrointestinal system [21]. In patients with diabetes mellitus, elevated blood glucose levels facilitate the occurrence of gut microbiota dysbiosis, thereby playing a role in the pathogenesis of DKD^4-7^. The gut-kidney axis delineates the interplay between the intestinal microbiome and renal pathologies, such as DKD. This interaction is reciprocal in nature. On one front, augmented uremic toxin levels in DKD modify the constitution and metabolic activities of the gut microbiota. Conversely, an imbalance in gut flora, known as dysbiosis, can compromise the integrity of the intestinal epithelial barrier, enhancing its permeability and facilitating greater systemic contact with bacterial endotoxins. This, in turn, triggers a cascade of noxious responses that can potentiate renal injury^8-12^. Emerging studies have shed light on disruption in this microbiota, leading to a deficiency of short-chain fatty acids (SCFAs), encompassing vital metabolites like propionate, acetate, and butyrate. These compounds are the by-products of healthy gut microbiota metabolic activities and have been notably linked to obesity, type 1 diabetes, and type 2 diabetes [22]. In the context of microbial metabolism, there is a discernible trend toward a reduction in saccharolytic microorganisms that are predominantly responsible for the synthesis of short-chain fatty acids (SCFAs), with a particular emphasis on butyrate-producing bacteria. The primary mode of action for SCFAs involves the activation of G-protein coupled receptors, such as GPR41, GPR43, and GPR109A, concurrently with the inhibition of histone deacetylase (HDAC)^13-16^. In individuals suffering from diabetic kidney disease (DKD), the engagement of G-protein coupled receptors (GPRs) by short-chain fatty acids (SCFAs) elicits an enhancement in the secretion of glucagon-like peptide-1 (GLP-1), which consequently enhances glucose tolerance and insulin sensitivity. With respect to the modulation of intestinal inflammation, SCFAs exert anti-inflammatory influences by upregulating the expression of the anti-inflammatory cytokine interleukin-10 (IL-10) while concurrently repressing the synthesis of pro-inflammatory cytokines (such as IL-6 and TNF-α) and inhibiting the activation of nuclear factor-κB (NF-κB). Moreover, sodium butyrate, a SCFA, has been observed to confer protective effects in DKD rat models, potentially through the stimulation of autophagic processes^17^. It is essential to note that DN stands as a major contributor to end-stage renal failure, closely intertwined with the conditions listed above. Furthermore, it appears that SCFAs play a pivotal role in reducing inflammation [23]. These SCFAs, stemming from the metabolite processes within the gut microbiota, have the power to influence kidney blood flow by activating the renin-angiotensin-system (RAAS), a system intricately associated with chronic kidney disease [24].

In our investigation, we unearthed a trio of microorganisms – Victivallaceae at the family level, *Eubacterium coprostanoligenes* and *Clostridium sensu stricto 1* – that exhibited a notably negative association with DN. This intriguing observation suggests a potentially protective role against the development of DN. It is worth noting that preceding observational studies have reported associations between heightened Clostridiaceae bacterial abundance and systemic inflammation, potentially elevating the risk of chronic kidney disease [25]. This somewhat contradicts our MR analysis findings, and the disparities could be attributed to confounding factors inherent in observational research that might exert an influence on the outcomes.

Alternatively, the chosen *p-*values during our experimentation might be a contributing factor. Thus, we undertook a Bonferroni correction, establishing significance thresholds for MR outcomes across five classification levels. The Bonferroni correction threshold for each classification level is denoted as 0.05/n, with ‘n’ representing the count of independent bacterial taxa at the corresponding classification level. A *p*-value below this Bonferroni correction threshold would signify a significant MR outcome. However, it is essential to underscore that these MR results failed to meet the stringent criteria set by Bonferroni’s multiple testing correction.

Conversely, among the remaining 12 affirmative findings, such as Bacteroidota, they uniformly point to a causal association with DN and an augmented risk of its occurrence. Pertinent studies have previously suggested that an upsurge in Bacteroidia abundance correlates with the severity of chronic kidney disease, possibly owing to the generation and accumulation of uremic toxins. Furthermore, this bacterial group has the capacity to activate the RAAS system by releasing inflammatory factors, aligning with the mechanisms outlined earlier in our analysis. On a related note, literature also indicates that specific bacterial groups, like *Allobaculum* and *Anaerosporobacter*, heighten the risk of developing diabetic kidney disease by amplifying the release of trimethylamine N-oxide (TMAO). Conversely, a surge in the abundance of Firmicutes appears to reduce the risk of DN. However, it is important to emphasize that this MR analysis did not uncover a causal link between these bacterial groups and DN.

Accounting for the pivotal role of gut microbiota dysbiosis in the advancement of DKD, remedial modalities targeting the intestinal microbiota are under active exploration for DKD management. These interventions encompass dietary adjustments, pharmacological agents, and fecal microbiota transplantation (FMT). Recent scientific inquiries have elucidated that dietary fiber contributes to the rectification of diabetes-induced microbial imbalance and affords protection against DKD development by facilitating the proliferation of SCFA producing bacteria, which are capable of diminishing inflammation and oxidative stress^18^. Regarding pharmacotherapy, an accumulating body of research documents that metformin enhances glucose homeostasis in DKD by elevating the prevalence of SCFA producing gut microbiota^19-22^. Similarly, Canagliflozin has been shown to diminish the accrual of uremic toxins and augment SCFA producing microbiota in a DKD murine model^23^. Moreover, a recent investigation has substantiated that empagliflozin mitigates DKD progression by modifying the gut microbiota profile, decreasing LPS producing bacteria while increasing SCFA producing bacteria^24^. Furthermore, the administration of healthy FMT has been identified as a modifier of the gut microbiome and a guardian against DKD exacerbation. Current data has positioned FMT as a safe and promising therapeutic approach for a spectrum of chronic disorders associated with alterations in gut microbiota, including inflammatory and immune system diseases^10,25^.

Our research has a few notable limitations that merit consideration. Firstly, the MR analysis was performed on a European population, and it remains unclear whether the outcomes can be generalized to represent the global population, encompassing a broader range of genetic and environmental diversities. Secondly, DN comprises five distinct stages, each characterized by varying levels of kidney impairment. The specific relationship between different gut microbiota and these diverse stages of DN has yet to be rigorously validated, warranting further research to explore this dimension. Thirdly, despite our establishment of a causal link between gut microbiota and DN, the precise mechanisms by which gut microbiota exert their influence on the development and progression of DN remain incompletely understood. This aspect constitutes an intriguing area for future research to unravel the underlying molecular and physiological pathways. Fourthly, our methodology employed a *p-*value threshold of <1 × 10^−5^ and employed a limited number of SNPs as instrumental variables. This strategy, while beneficial in maintaining statistical rigor, may have some limitations in explaining a broader spectrum of exposure variation, potentially affecting the statistical power of causal estimation. Future research endeavors might benefit from exploring additional SNPs and employing different threshold criteria to gain a more comprehensive understanding of the causal associations underpinning the role of gut microbiota in DN.

## Conclusion

By employing Mendelian Randomization (MR) analysis, our investigation has successfully validated a causal association between the gut microbiota and diabetic nephropathy. The outcomes of our comprehensive MR analysis present novel perspectives that hold the potential to revolutionize diagnostic methods and therapeutic interventions, particularly concerning the treatment of diabetic nephropathy based on the principles of gut microecology.

## Supplementary Material

Supplementary Table S1.docx

Supplementary Table S2.docx

Supplementary Table S3.xlsx

## Data Availability

This study relies on publicly accessible datasets for analysis. The GWAS summary data for DN can be obtained from the Finngen website (https://www.finngen.fi/en). Additionally, the summary statistics pertaining to microbial features are accessible through the MiBioGen cohort at www.mibiogen.org.

## References

[1] Gill D, Efstathiadou A, Cawood K, et al. Education protects against coronary heart disease and stroke independently of cognitive function: evidence from Mendelian randomization. Int J Epidemiol. 2019;48(5):1468–1477. doi: 10.1093/ije/dyz200. 31562522 PMC6857750

[2] Levin MG, Judy R, Gill D, et al. Genetics of height and risk of atrial fibrillation: a Mendelian randomization study. PLoS Med. 2020;17(10):e1003288. doi: 10.1371/journal.pmed.1003288. 33031386 PMC7544133

[3] Palmer TM, Lawlor DA, Harbord RM, et al. Using multiple genetic variants as instrumental variables for modifiable risk factors. Stat Methods Med Res. 2012;21(3):223–242. doi: 10.1177/0962280210394459. 21216802 PMC3917707

[4] Fernandes R, Viana SD, Nunes S, et al. Diabetic gut microbiota dysbiosis as an inflammaging and immunosenescence condition that fosters progression of retinopathy and nephropathy. Biochim Biophys Acta Mol Basis Dis. 2019;1865(7):1876–1897. doi: 10.1016/j.bbadis.2018.09.032. 30287404

[5] Iatcu CO, Steen A, Covasa M. Gut Microbiota and Complications of Type-2 Diabetes. Nutrients. 2021;14(1):166. doi: 10.3390/nu14010166. 35011044 PMC8747253

[6] Mosterd CM, Kanbay M, van den Born BJH, et al. Intestinal microbiota and diabetic kidney diseases: the Role of microbiota and derived metabolites inmodulation of renal inflammation and disease progression. Best Pract Res Clin Endocrinol Metab. 2021;35(3):101484. doi: 10.1016/j.beem.2021.101484. 33546983

[7] Nagase N, Ikeda Y, Tsuji A, et al. Efficacy of probiotics on the modulation of gut microbiota in the treatment of diabetic nephropathy. World J Diabetes. 2022;13(3):150–160. doi: 10.4239/wjd.v13.i3.150. 35432750 PMC8984564

[8] Cai T-T, Ye X-L, Li R-R, et al. Resveratrol modulates the gut microbiota and inflammation to protect against diabetic nephropathy in mice. Front Pharmacol. 2020;11:1249. doi: 10.3389/fphar.2020.01249. 32973502 PMC7466761

[9] Evenepoel P, Poesen R, Meijers B. The gut-kidney axis. Pediatr Nephrol. 2017;32(11):2005–2014. doi: 10.1007/s00467-016-3527-x. 27848096

[10] Ni Y, Zheng L, Nan S, et al. Enterorenal crosstalks in diabetic nephropathy and novel therapeutics targeting the gut microbiota. Acta Biochim Biophys Sin (Shanghai). 2022;54(10):1406–1420. doi: 10.3724/abbs.2022140. 36239349 PMC9827797

[11] Wang P, Wang T, Zheng X, et al. Gut microbiota, key to unlocking the door of diabetic kidney disease. Nephrology (Carlton). 2021;26(8):641–649. doi: 10.1111/nep.13874. 33715272 PMC8360003

[12] Zhang Q, Xiao X, Li M, et al. Vildagliptin increases butyrate-producing bacteria in the gut of diabetic rats. PLoS One. 2017;12(10):e0184735. doi: 10.1371/journal.pone.0184735. 29036231 PMC5643055

[13] Andrade-Oliveira V, Amano MT, Correa-Costa M, et al. Gut Bacteria Products Prevent AKI Induced by Ischemia-Reperfusion. J Am Soc Nephrol. 2015;26(8):1877–1888. doi: 10.1681/asn.2014030288. 25589612 PMC4520159

[14] Cheng X, Zhou T, He Y, et al. The role and mechanism of butyrate in the prevention and treatment of diabetic kidney disease. Front Microbiol. 2022;13:961536. doi: 10.3389/fmicb.2022.961536. 36016798 PMC9396028

[15] Fang Q, Liu N, Zheng B, et al. Roles of Gut Microbial Metabolites in Diabetic Kidney Disease. Front Endocrinol (Lausanne). 2021;12:636175. doi: 10.3389/fendo.2021.636175. 34093430 PMC8173181

[16] Lin J-R, Wang Z-T, Sun J-J, et al. Gut microbiota and diabetic kidney diseases: pathogenesis and therapeutic perspectives. World J Diabetes. 2022;13(4):308–318. doi: 10.4239/wjd.v13.i4.308. 35582668 PMC9052008

[17] Kim CH, Park J, Kim M. Gut microbiota-derived short-chain Fatty acids, T cells, and inflammation. Immune Netw. 2014;14(6):277–288. doi: 10.4110/in.2014.14.6.277. 25550694 PMC4275385

[18] Drake AM, Coughlan MT, Christophersen CT, et al. Resistant Starch as a Dietary Intervention to Limit the Progression of Diabetic Kidney Disease. Nutrients. 2022;14(21):4547. doi: 10.3390/nu14214547. 36364808 PMC9656781

[19] de la Cuesta-Zuluaga J, Mueller NT, Corrales-Agudelo V, et al. Metformin is associated with higher relative abundance of mucin-degrading akkermansia muciniphila and several short-chain fatty acid-producing microbiota in the gut. Diabetes Care. 2017;40(1):54–62. doi: 10.2337/dc16-1324. 27999002

[20] Lee CB, Chae SU, Jo SJ, et al. The relationship between the gut microbiome and metformin as a key for treating type 2 diabetes mellitus. Int J Mol Sci. 2021;22(7):3566. doi: 10.3390/ijms22073566. PMC8037857 33808194

[21] Sun L, Xie C, Wang G, et al. Gut microbiota and intestinal FXR mediate the clinical benefits of metformin. Nat Med. 2018;24(12):1919–1929. doi: 10.1038/s41591-018-0222-4. 30397356 PMC6479226

[22] Vallianou NG, Stratigou T, Tsagarakis S. Metformin and gut microbiota: their interactions and their impact on diabetes. Hormones (Athens). 2019;18(2):141–144. doi: 10.1007/s42000-019-00093-w. 30719628

[23] Mishima E, Fukuda S, Kanemitsu Y, et al. Canagliflozin reduces plasma uremic toxins and alters the intestinal microbiota composition in a chronic kidney disease mouse model. Am J Physiol Renal Physiol. 2018;315(4):F824–f833. doi: 10.1152/ajprenal.00314.2017. 29167170

[24] Deng L, Yang Y, Xu G. Empagliflozin ameliorates type 2 diabetes mellitus-related diabetic nephropathy via altering the gut microbiota. Biochim Biophys Acta Mol Cell Biol Lipids. 2022;1867(12):159234. (([0-9][0-9][0-9][0-9])). doi: 10.1016/j.bbalip.2022.159234. 36185030

[25] Danne C, Rolhion N, Sokol H. Recipient factors in faecal microbiota transplantation: one stool does not fit all. Nat Rev Gastroenterol Hepatol. 2021;18(7):503–513. doi: 10.1038/s41575-021-00441-5. 33907321

