# Supplementary material for: Exploring the causal connection: insights into diabetic nephropathy and gut microbiota from whole-genome sequencing databases
Source: Ren Fail. 2024 Aug 1;46(2):2385065. doi: 10.1080/0886022X.2024.2385065 (PMC11299436; doi:10.1080/0886022X.2024.2385065)
Supplement: Supplementary Table S1.docx [file IRNF_A_2385065_SM7171.docx]

Supplementary Table S1. SNPs were used as instrumental variables from gut microbiome and DN GWASs (P <1 ×10^-5^).

| Name | N | SNP | CHR | Position | beta. exposure | se. exposure | beta. outcome | se. outcome | F |
| --- | --- | --- | --- | --- | --- | --- | --- | --- | --- |
| class.Bacteroidia | 1 | rs11146701 | 10 | 39062269 | 0.047359582 | 0.010540996 | -0.00878385 | 0.0236857 | 20.18388436 |
|  | 2 | rs13291169 | 9 | 116888284 | 0.068967579 | 0.014876501 | 0.000201505 | 0.0324615 | 21.49011655 |
|  | 3 | rs17343978 | 22 | 27433885 | -0.055205684 | 0.012027767 | -0.0354087 | 0.0278897 | 21.06445364 |
|  | 4 | rs2032750 | 2 | 53831026 | -0.050839276 | 0.010680731 | -0.0196334 | 0.0225926 | 22.65416737 |
|  | 5 | rs4916508 | 3 | 195936789 | -0.046672941 | 0.010531247 | -0.0236171 | 0.0226713 | 19.63917924 |
|  | 6 | rs55773148 | 13 | 70523029 | -0.121513597 | 0.023675903 | -0.0810269 | 0.0497028 | 26.33662431 |
|  | 7 | rs62531359 | 8 | 70916181 | 0.065572807 | 0.014989058 | 0.0187324 | 0.029414 | 19.13601291 |
|  | 8 | rs62575403 | 9 | 136493820 | 0.140054866 | 0.031112824 | -0.00746234 | 0.0560935 | 20.25945178 |
|  | 9 | rs72706335 | 1 | 157495438 | -0.222409754 | 0.049345816 | -0.143674 | 0.0730056 | 20.30390118 |
|  | 10 | rs73975615 | 17 | 6461200 | -0.207017892 | 0.044263343 | 0.0508691 | 0.13195 | 21.86089731 |
|  | 11 | rs7546249 | 1 | 176792690 | -0.056711256 | 0.01183859 | -0.0229617 | 0.0246572 | 22.94516528 |
|  | 12 | rs7631304 | 3 | 89339527 | -0.064577053 | 0.013295739 | -0.0110738 | 0.0318068 | 23.58761894 |
|  | 13 | rs79585701 | 8 | 13110185 | 0.064682288 | 0.01496282 | 0.0316277 | 0.0322646 | 18.68514473 |
|  | 14 | rs929878 | 16 | 74290641 | -0.054853206 | 0.012152954 | -0.0364091 | 0.0275219 | 20.37008987 |
| class.Verrucomicrobiae | 1 | rs11184341 | 1 | 105422565 | 0.065534193 | 0.014223078 | 0.0539605 | 0.0251841 | 21.22635923 |
|  | 2 | rs111862613 | 12 | 130309670 | 0.090698957 | 0.019674596 | 0.0465871 | 0.0301662 | 21.24800646 |
|  | 3 | rs117107102 | 18 | 49473635 | 0.20468288 | 0.043157251 | -0.00819176 | 0.0534736 | 22.48437735 |
|  | 4 | rs11729256 | 4 | 95027272 | 0.074979802 | 0.015017677 | 0.0307179 | 0.0299945 | 24.92354551 |
|  | 5 | rs12908520 | 15 | 97570657 | 0.061892558 | 0.013094626 | 0.0141422 | 0.0226745 | 22.3366193 |
|  | 6 | rs2602429 | 16 | 81063149 | 0.074684437 | 0.015619438 | 0.0478029 | 0.0257148 | 22.85888841 |
|  | 7 | rs4242783 | 10 | 5064327 | 0.068929219 | 0.014769297 | 0.0611826 | 0.0250924 | 21.77773754 |
|  | 8 | rs4936098 | 11 | 130280667 | 0.064884253 | 0.013592809 | 0.0418097 | 0.0235929 | 22.78174224 |
|  | 9 | rs61779207 | 1 | 41074472 | -0.075830853 | 0.016776157 | -0.0139326 | 0.0272469 | 20.42828819 |
|  | 10 | rs74542928 | 4 | 100544188 | 0.112163707 | 0.023642195 | 0.0642098 | 0.0523867 | 22.50358289 |
|  | 11 | rs9349825 | 6 | 56341481 | -0.070403566 | 0.014712789 | 0.0273913 | 0.0286732 | 22.89423021 |
|  | 12 | rs941682 | 20 | 31867840 | -0.063143598 | 0.014376741 | -0.0189144 | 0.0251219 | 19.28697833 |
| family.Peptostreptococcaceae | 1 | rs10805326 | 4 | 14324623 | 0.0566637 | 0.012275984 | 0.00627214 | 0.0247056 | 21.30311675 |
|  | 2 | rs117020988 | 7 | 46710977 | 0.182411122 | 0.037212625 | 0.0457393 | 0.0415079 | 24.021208 |
|  | 3 | rs12377846 | 9 | 16786784 | -0.252000426 | 0.051162723 | -0.103846 | 0.0690412 | 24.24726142 |
|  | 4 | rs12986312 | 19 | 17414138 | 0.057181848 | 0.01259472 | 0.0061464 | 0.0245958 | 20.6103301 |
|  | 5 | rs1467258 | 17 | 38216487 | 0.072583976 | 0.016232118 | -0.0138224 | 0.0291437 | 19.99288126 |
|  | 6 | rs1520207 | 3 | 151781175 | 0.052565835 | 0.011247608 | -0.00514276 | 0.0225472 | 21.83899725 |
|  | 7 | rs2644627 | 8 | 26726641 | -0.055850562 | 0.011224002 | -0.0340165 | 0.0227865 | 24.75742851 |
|  | 8 | rs4692811 | 4 | 171180190 | 0.064207491 | 0.012691273 | 0.0255313 | 0.0237343 | 25.59216002 |
|  | 9 | rs59865771 | 16 | 89246670 | -0.057376395 | 0.012719207 | -0.0324661 | 0.0237172 | 20.34662074 |
|  | 10 | rs61841503 | 10 | 17019559 | 0.091965839 | 0.016133103 | 0.0175817 | 0.0335747 | 32.49081531 |
|  | 11 | rs6721459 | 2 | 67852836 | 0.050982344 | 0.011197512 | 0.0220091 | 0.0228788 | 20.72728605 |
|  | 12 | rs75819860 | 18 | 65324280 | 0.126263781 | 0.026838281 | 0.029671 | 0.0362984 | 22.12961841 |
|  | 13 | rs76982728 | 7 | 44312824 | 0.124202832 | 0.026692325 | -0.129324 | 0.0782901 | 21.64773811 |
|  | 14 | rs77540684 | 10 | 14650535 | 0.106725055 | 0.024563751 | -0.0162441 | 0.0383641 | 18.87465549 |
|  | 15 | rs9573937 | 13 | 77351196 | -0.069363688 | 0.01429074 | -0.010668 | 0.0303697 | 23.55596131 |
| family.Veillonellaceae | 1 | rs111810795 | 14 | 102897930 | -0.08671084 | 0.018057347 | -0.00455102 | 0.0379924 | 23.05596132 |
|  | 2 | rs114889439 | 13 | 61597625 | -0.253809201 | 0.053916917 | -0.112025 | 0.0614283 | 22.14617637 |
|  | 3 | rs12186441 | 5 | 132646303 | 0.208095725 | 0.045399862 | -0.00848078 | 0.0551914 | 21.00154918 |
|  | 4 | rs12668619 | 7 | 21637810 | 0.055300975 | 0.011765373 | 0.00777141 | 0.0239457 | 22.09020709 |
|  | 5 | rs12741784 | 1 | 50088819 | -0.062145234 | 0.011926612 | -0.0217336 | 0.0256531 | 27.14730537 |
|  | 6 | rs1442060 | 4 | 46366067 | -0.051402432 | 0.011212452 | 0.0283454 | 0.0226685 | 21.01414715 |
|  | 7 | rs1447205 | 8 | 41231597 | -0.052068875 | 0.011565567 | -0.0375126 | 0.0234534 | 20.26600485 |
|  | 8 | rs1693340 | 18 | 30208691 | 0.081893428 | 0.018245018 | -0.0131142 | 0.0437585 | 20.14428339 |
|  | 9 | rs2175069 | 4 | 23317124 | -0.052675165 | 0.01148863 | -0.0204631 | 0.0232088 | 21.01943083 |
|  | 10 | rs2561116 | 5 | 38348199 | -0.083616969 | 0.018715906 | -0.0350143 | 0.0482978 | 19.95780429 |
|  | 11 | rs2585520 | 13 | 78785141 | -0.090497192 | 0.020042803 | 0.0178587 | 0.0579535 | 20.38436814 |
|  | 12 | rs4263802 | 8 | 138319508 | 0.050941954 | 0.011492112 | -0.000201801 | 0.0238978 | 19.6470399 |
|  | 13 | rs4461038 | 15 | 78546996 | -0.055496494 | 0.011946736 | -0.0078579 | 0.0247834 | 21.57635998 |
|  | 14 | rs4797169 | 18 | 462180 | 0.058779888 | 0.012829669 | 0.0112855 | 0.0264214 | 20.98805657 |
|  | 15 | rs61264131 | 6 | 139188534 | 0.202366989 | 0.046438799 | 0.0553833 | 0.0382006 | 18.97731738 |
|  | 16 | rs6692542 | 1 | 244391618 | 0.053439722 | 0.011825371 | 0.0189978 | 0.0235313 | 20.41946969 |
|  | 17 | rs6909981 | 6 | 75534152 | -0.063869651 | 0.014176514 | 0.0453898 | 0.0339686 | 20.29531574 |
|  | 18 | rs75768969 | 13 | 101617913 | -0.070377296 | 0.015685477 | -0.00279901 | 0.0401626 | 20.12868101 |
|  | 19 | rs79535861 | 13 | 38326692 | 0.100666153 | 0.020701553 | 0.0991479 | 0.0393094 | 23.64304536 |
|  | 20 | rs9345168 | 6 | 92380922 | 0.050910225 | 0.011325295 | -0.0328321 | 0.0225681 | 20.20489577 |
| family.Verrucomicrobiaceae | 1 | rs11184341 | 1 | 105422565 | 0.065530075 | 0.014223048 | 0.0539605 | 0.0251841 | 21.22378123 |
|  | 2 | rs111862613 | 12 | 130309670 | 0.090706497 | 0.01967463 | 0.0465871 | 0.0301662 | 21.25146594 |
|  | 3 | rs117107102 | 18 | 49473635 | 0.20468288 | 0.043157251 | -0.00819176 | 0.0534736 | 22.48437735 |
|  | 4 | rs11729256 | 4 | 95027272 | 0.074979802 | 0.015017677 | 0.0307179 | 0.0299945 | 24.92354551 |
|  | 5 | rs12908520 | 15 | 97570657 | 0.061910394 | 0.013094609 | 0.0141422 | 0.0226745 | 22.34955298 |
|  | 6 | rs2602429 | 16 | 81063149 | 0.074551564 | 0.015619484 | 0.0478029 | 0.0257148 | 22.77748893 |
|  | 7 | rs4242783 | 10 | 5064327 | 0.068799208 | 0.01476936 | 0.0611826 | 0.0250924 | 21.69547767 |
|  | 8 | rs4936098 | 11 | 130280667 | 0.0648691 | 0.013592808 | 0.0418097 | 0.0235929 | 22.77110599 |
|  | 9 | rs61779207 | 1 | 41074472 | -0.075881119 | 0.01677619 | -0.0139326 | 0.0272469 | 20.45529929 |
|  | 10 | rs74542928 | 4 | 100544188 | 0.112123956 | 0.023642178 | 0.0642098 | 0.0523867 | 22.48766745 |
|  | 11 | rs9349825 | 6 | 56341481 | -0.070435405 | 0.014712796 | 0.0273913 | 0.0286732 | 22.91492026 |
|  | 12 | rs941682 | 20 | 31867840 | -0.06315257 | 0.014376725 | -0.0189144 | 0.0251219 | 19.29250259 |
| family.Victivallaceae | 1 | rs11671100 | 19 | 711637 | -0.160034432 | 0.034947236 | -0.00323838 | 0.027928 | 20.95754283 |
|  | 2 | rs11764871 | 7 | 146808977 | 0.12709902 | 0.025655983 | -0.00200801 | 0.0244441 | 24.52928145 |
|  | 3 | rs2546105 | 5 | 94692439 | 0.126643495 | 0.025711059 | -0.0193308 | 0.0261004 | 24.24956987 |
|  | 4 | rs2944282 | 7 | 57589803 | -0.124196623 | 0.025702577 | 0.0572018 | 0.0251236 | 23.33575406 |
|  | 5 | rs34962571 | 12 | 131103256 | -0.186945459 | 0.04184118 | 0.0461844 | 0.0376113 | 19.95049054 |
|  | 6 | rs4396289 | 11 | 9316730 | -0.152917575 | 0.028852532 | 0.0182874 | 0.033211 | 28.0753326 |
|  | 7 | rs61702987 | 2 | 31211657 | 0.145501293 | 0.030000733 | -0.000892671 | 0.0360666 | 23.50972471 |
|  | 8 | rs62570196 | 9 | 111086170 | -0.246101492 | 0.048307716 | 0.044277 | 0.0557232 | 25.93668023 |
|  | 9 | rs6545794 | 2 | 60484909 | -0.19778562 | 0.041049541 | 0.00138799 | 0.0344894 | 23.20336403 |
|  | 10 | rs7077363 | 10 | 95286954 | 0.14894216 | 0.031836622 | -0.0474067 | 0.0305628 | 21.87556329 |
|  | 11 | rs7314815 | 12 | 102525047 | 0.101087856 | 0.02255936 | 0.00648724 | 0.0226385 | 20.06884289 |
|  | 12 | rs7627405 | 3 | 9968555 | -0.134158069 | 0.030142747 | 0.0408267 | 0.0283166 | 19.79810134 |
|  | 13 | rs7860510 | 9 | 94128796 | 0.164855226 | 0.037512763 | -0.0112707 | 0.0422375 | 19.30301921 |
| genus..Eubacteriumcoprostanoligenesgroup | 1 | rs1020520 | 7 | 33603146 | -0.059070472 | 0.013292872 | -0.0117931 | 0.031405 | 19.74482238 |
|  | 2 | rs10444197 | 10 | 2215931 | -0.050582278 | 0.011345529 | -0.00988065 | 0.0236148 | 19.87454929 |
|  | 3 | rs11052069 | 12 | 32713919 | 0.047783175 | 0.01078333 | -0.0347619 | 0.0225991 | 19.63334218 |
|  | 4 | rs11720857 | 3 | 113794507 | 0.063077578 | 0.014448482 | -0.0348323 | 0.0293715 | 19.05699233 |
|  | 5 | rs12906958 | 15 | 36911598 | -0.053315925 | 0.011593477 | 0.0187341 | 0.0247252 | 21.14640317 |
|  | 6 | rs17159861 | 7 | 31085162 | 0.096222758 | 0.016838828 | -0.0921304 | 0.0364384 | 32.64990339 |
|  | 7 | rs2644213 | 10 | 84506096 | 0.053884526 | 0.012124458 | -0.0301197 | 0.0245446 | 19.74938098 |
|  | 8 | rs4076415 | 15 | 86440997 | 0.051515202 | 0.011029435 | 0.00702699 | 0.0233191 | 21.81288281 |
|  | 9 | rs4717831 | 7 | 73306506 | 0.078678267 | 0.017403801 | -0.0107945 | 0.0320752 | 20.43477283 |
|  | 10 | rs62024432 | 15 | 97869293 | -0.076969763 | 0.017210135 | 0.052219 | 0.0384439 | 19.99960646 |
|  | 11 | rs6762473 | 3 | 127139574 | 0.052158365 | 0.011235251 | 0.0384645 | 0.0236705 | 21.54926154 |
|  | 12 | rs76898927 | 3 | 81595477 | 0.12305502 | 0.026637491 | -0.0340544 | 0.0494507 | 21.33736663 |
|  | 13 | rs9648214 | 7 | 16380806 | -0.082871942 | 0.016430987 | 0.0176773 | 0.0399958 | 25.43535164 |
| genus.Akkermansia | 1 | rs11184341 | 1 | 105422565 | 0.065585479 | 0.01422364 | 0.0539605 | 0.0251841 | 21.25791477 |
|  | 2 | rs111862613 | 12 | 130309670 | 0.091119928 | 0.019674815 | 0.0465871 | 0.0301662 | 21.44522786 |
|  | 3 | rs117107102 | 18 | 49473635 | 0.204406166 | 0.043162894 | -0.00819176 | 0.0534736 | 22.41775984 |
|  | 4 | rs11729256 | 4 | 95027272 | 0.075047253 | 0.015018395 | 0.0307179 | 0.0299945 | 24.96601989 |
|  | 5 | rs12908520 | 15 | 97570657 | 0.061772023 | 0.01309539 | 0.0141422 | 0.0226745 | 22.24710705 |
|  | 6 | rs2602429 | 16 | 81063149 | 0.074535241 | 0.015620127 | 0.0478029 | 0.0257148 | 22.76564107 |
|  | 7 | rs4242783 | 10 | 5064327 | 0.068545381 | 0.014770062 | 0.0611826 | 0.0250924 | 21.5336395 |
|  | 8 | rs4936098 | 11 | 130280667 | 0.064922492 | 0.01359338 | 0.0418097 | 0.0235929 | 22.80668615 |
|  | 9 | rs61779207 | 1 | 41074472 | -0.076053856 | 0.01677691 | -0.0139326 | 0.0272469 | 20.54677084 |
|  | 10 | rs74542928 | 4 | 100544188 | 0.112622737 | 0.023643438 | 0.0642098 | 0.0523867 | 22.68576578 |
|  | 11 | rs9349825 | 6 | 56341481 | -0.070340692 | 0.014713291 | 0.0273913 | 0.0286732 | 22.85179726 |
|  | 12 | rs941682 | 20 | 31867840 | -0.063295745 | 0.01437768 | -0.0189144 | 0.0251219 | 19.37750419 |
| genus.Catenibacterium | 1 | rs12404911 | 1 | 240118443 | 0.140717216 | 0.030414222 | 0.0413115 | 0.0288036 | 21.39305446 |
|  | 2 | rs212393 | 6 | 159485742 | -0.135253856 | 0.02862088 | -0.0419074 | 0.0277388 | 22.31850482 |
|  | 3 | rs73128290 | 7 | 57364320 | 0.129726249 | 0.02845626 | 0.0435481 | 0.0245759 | 20.76977694 |
|  | 4 | rs7742829 | 6 | 105089665 | 0.114110133 | 0.025109895 | 0.0164392 | 0.022611 | 20.63908222 |
| genus.Clostridiumsensustricto1 | 1 | rs11264403 | 1 | 155676492 | -0.139061503 | 0.033445425 | -0.0332493 | 0.0429212 | 17.28308054 |
|  | 2 | rs115807074 | 5 | 84997131 | 0.110966819 | 0.025049788 | -0.0975727 | 0.0625286 | 21.2949551 |
|  | 3 | rs116847295 | 12 | 43485015 | 0.110021418 | 0.024603155 | -0.0529948 | 0.03384 | 19.9942715 |
|  | 4 | rs12341505 | 9 | 136710881 | 0.081071775 | 0.018012111 | 0.000686411 | 0.0385715 | 20.25576296 |
|  | 5 | rs12490337 | 3 | 111482845 | -0.061687867 | 0.0137581 | 0.0145436 | 0.0280931 | 20.10115763 |
|  | 6 | rs2795528 | 10 | 43270264 | -0.184314695 | 0.039220419 | 0.0551159 | 0.0491042 | 22.07796307 |
|  | 7 | rs2817172 | 1 | 3041519 | 0.058138957 | 0.012449219 | -0.0289464 | 0.0229646 | 21.80653479 |
|  | 8 | rs550843 | 6 | 165722832 | -0.078324645 | 0.016920943 | 0.0289506 | 0.025164 | 21.42328906 |
| genus.Lachnoclostridium | 1 | rs1031599 | 3 | 66725825 | -0.078627049 | 0.017564435 | -0.0163504 | 0.0456556 | 20.03667511 |
|  | 2 | rs12566975 | 1 | 185091351 | -0.04680969 | 0.010578675 | -0.0144512 | 0.0225313 | 19.57764774 |
|  | 3 | rs1528479 | 2 | 167244051 | -0.049779849 | 0.011191926 | -0.0298824 | 0.0232651 | 19.78097928 |
|  | 4 | rs1801968 | 9 | 132580901 | -0.068914709 | 0.01583026 | -0.0455331 | 0.0307943 | 18.94945003 |
|  | 5 | rs1997204 | 12 | 102046595 | -0.108074801 | 0.024202203 | -0.00110308 | 0.0544113 | 19.93781902 |
|  | 6 | rs2385421 | 18 | 19743455 | 0.074618572 | 0.018073408 | 0.0106172 | 0.0347744 | 17.04375342 |
|  | 7 | rs3821998 | 4 | 38694566 | -0.0864066 | 0.019251946 | -0.0668574 | 0.0367729 | 20.14169847 |
|  | 8 | rs4738679 | 8 | 59370320 | -0.052026675 | 0.011404049 | -0.0346409 | 0.0231067 | 20.81064413 |
|  | 9 | rs6112314 | 20 | 19300846 | -0.056171476 | 0.010817419 | -0.0375389 | 0.0237689 | 26.96098053 |
|  | 10 | rs615997 | 3 | 23037786 | 0.05117524 | 0.010649056 | -0.0274758 | 0.022426 | 23.0913358 |
|  | 11 | rs61915992 | 12 | 28169306 | 0.080387577 | 0.01722087 | -0.00439655 | 0.0321037 | 21.78792705 |
|  | 12 | rs62028349 | 16 | 27217620 | 0.046998893 | 0.010597086 | -0.00506712 | 0.0226837 | 19.66771345 |
|  | 13 | rs62285313 | 3 | 177470032 | 0.086420325 | 0.018156544 | 0.0623725 | 0.0380974 | 22.65240376 |
|  | 14 | rs72829893 | 17 | 46694541 | 0.117472377 | 0.026810315 | 0.0154329 | 0.0369988 | 19.19542134 |
|  | 15 | rs78068103 | 17 | 13816159 | 0.088619929 | 0.019424795 | 0.0325899 | 0.0352178 | 20.81134771 |
|  | 16 | rs789029 | 18 | 1053252 | -0.06412885 | 0.013797406 | -0.0474783 | 0.0323662 | 21.60048728 |
| genus.Parasutterella | 1 | rs10899911 | 10 | 44293839 | -0.071710162 | 0.014815144 | -0.0358332 | 0.0264939 | 23.42466349 |
|  | 2 | rs11715853 | 3 | 30163689 | -0.066295189 | 0.014611398 | 0.00321292 | 0.0246941 | 20.5827876 |
|  | 3 | rs1403396 | 12 | 126498824 | -0.075672871 | 0.015903462 | -0.0116459 | 0.027577 | 22.63709667 |
|  | 4 | rs2090816 | 6 | 137615592 | 0.084096728 | 0.017731494 | 0.0246107 | 0.0291671 | 22.49009239 |
|  | 5 | rs35055552 | 8 | 114804024 | 0.109554176 | 0.023543268 | 0.027718 | 0.0327045 | 21.64897065 |
|  | 6 | rs35414597 | 4 | 15565736 | -0.068494619 | 0.01421433 | -0.00849004 | 0.0245036 | 23.21580934 |
|  | 7 | rs55877868 | 17 | 14692867 | -0.10445781 | 0.022808932 | -0.000184071 | 0.0375354 | 20.96958758 |
|  | 8 | rs62273907 | 3 | 156550415 | 0.229468131 | 0.050226185 | 0.00503648 | 0.0448525 | 20.86166575 |
|  | 9 | rs6809952 | 3 | 193896709 | -0.068495728 | 0.015089156 | -0.00301711 | 0.0254363 | 20.6024398 |
|  | 10 | rs6828768 | 4 | 65556925 | 0.063685185 | 0.013264516 | 0.016441 | 0.0224868 | 23.04717615 |
|  | 11 | rs7303158 | 12 | 5275540 | 0.064685912 | 0.013425592 | 0.0117596 | 0.0226 | 23.21005817 |
|  | 12 | rs7311004 | 12 | 53260710 | -0.061752212 | 0.01364384 | -0.0251588 | 0.0226819 | 20.48120461 |
|  | 13 | rs7572229 | 2 | 72235444 | 0.066272974 | 0.013273581 | 0.0399211 | 0.0225876 | 24.92415244 |
|  | 14 | rs78383039 | 2 | 178954708 | -0.146314647 | 0.029711928 | -0.0961488 | 0.0575064 | 24.24483596 |
|  | 15 | rs8039785 | 15 | 67316307 | 0.061834541 | 0.013300041 | 0.018054 | 0.0224896 | 21.61125614 |
|  | 16 | rs823424 | 8 | 16674526 | -0.071344819 | 0.015695821 | -0.0299238 | 0.0258501 | 20.65763093 |
| order.Bacteroidales | 1 | rs11146701 | 10 | 39062269 | 0.047359582 | 0.010540996 | -0.00878385 | 0.0236857 | 20.18388436 |
|  | 2 | rs13291169 | 9 | 116888284 | 0.068967579 | 0.014876501 | 0.000201505 | 0.0324615 | 21.49011655 |
|  | 3 | rs17343978 | 22 | 27433885 | -0.055205684 | 0.012027767 | -0.0354087 | 0.0278897 | 21.06445364 |
|  | 4 | rs2032750 | 2 | 53831026 | -0.050839276 | 0.010680731 | -0.0196334 | 0.0225926 | 22.65416737 |
|  | 5 | rs4916508 | 3 | 195936789 | -0.046672941 | 0.010531247 | -0.0236171 | 0.0226713 | 19.63917924 |
|  | 6 | rs55773148 | 13 | 70523029 | -0.121513597 | 0.023675903 | -0.0810269 | 0.0497028 | 26.33662431 |
|  | 7 | rs62531359 | 8 | 70916181 | 0.065572807 | 0.014989058 | 0.0187324 | 0.029414 | 19.13601291 |
|  | 8 | rs62575403 | 9 | 136493820 | 0.140054866 | 0.031112824 | -0.00746234 | 0.0560935 | 20.25945178 |
|  | 9 | rs72706335 | 1 | 157495438 | -0.222409754 | 0.049345816 | -0.143674 | 0.0730056 | 20.30390118 |
|  | 10 | rs73975615 | 17 | 6461200 | -0.207017892 | 0.044263343 | 0.0508691 | 0.13195 | 21.86089731 |
|  | 11 | rs7546249 | 1 | 176792690 | -0.056711256 | 0.01183859 | -0.0229617 | 0.0246572 | 22.94516528 |
|  | 12 | rs7631304 | 3 | 89339527 | -0.064577053 | 0.013295739 | -0.0110738 | 0.0318068 | 23.58761894 |
|  | 13 | rs79585701 | 8 | 13110185 | 0.064682288 | 0.01496282 | 0.0316277 | 0.0322646 | 18.68514473 |
|  | 14 | rs929878 | 16 | 74290641 | -0.054853206 | 0.012152954 | -0.0364091 | 0.0275219 | 20.37008987 |
| order.Verrucomicrobiales | 1 | rs11184341 | 1 | 105422565 | 0.065534193 | 0.014223078 | 0.0539605 | 0.0251841 | 21.22635923 |
|  | 2 | rs111862613 | 12 | 130309670 | 0.090698957 | 0.019674596 | 0.0465871 | 0.0301662 | 21.24800646 |
|  | 3 | rs117107102 | 18 | 49473635 | 0.20468288 | 0.043157251 | -0.00819176 | 0.0534736 | 22.48437735 |
|  | 4 | rs11729256 | 4 | 95027272 | 0.074979802 | 0.015017677 | 0.0307179 | 0.0299945 | 24.92354551 |
|  | 5 | rs12908520 | 15 | 97570657 | 0.061892558 | 0.013094626 | 0.0141422 | 0.0226745 | 22.3366193 |
|  | 6 | rs2602429 | 16 | 81063149 | 0.074684437 | 0.015619438 | 0.0478029 | 0.0257148 | 22.85888841 |
|  | 7 | rs4242783 | 10 | 5064327 | 0.068929219 | 0.014769297 | 0.0611826 | 0.0250924 | 21.77773754 |
|  | 8 | rs4936098 | 11 | 130280667 | 0.064884253 | 0.013592809 | 0.0418097 | 0.0235929 | 22.78174224 |
|  | 9 | rs61779207 | 1 | 41074472 | -0.075830853 | 0.016776157 | -0.0139326 | 0.0272469 | 20.42828819 |
|  | 10 | rs74542928 | 4 | 100544188 | 0.112163707 | 0.023642195 | 0.0642098 | 0.0523867 | 22.50358289 |
|  | 11 | rs9349825 | 6 | 56341481 | -0.070403566 | 0.014712789 | 0.0273913 | 0.0286732 | 22.89423021 |
|  | 12 | rs941682 | 20 | 31867840 | -0.063143598 | 0.014376741 | -0.0189144 | 0.0251219 | 19.28697833 |
| phylum.Bacteroidetes | 1 | rs13291169 | 9 | 116888284 | 0.070092873 | 0.014875617 | 0.000201505 | 0.0324615 | 22.19975338 |
|  | 2 | rs17343978 | 22 | 27433885 | -0.055604102 | 0.012027223 | -0.0354087 | 0.0278897 | 21.37152731 |
|  | 3 | rs2032750 | 2 | 53831026 | -0.051085659 | 0.010680423 | -0.0196334 | 0.0225926 | 22.87559723 |
|  | 4 | rs62531359 | 8 | 70916181 | 0.06577884 | 0.014988611 | 0.0187324 | 0.029414 | 19.25760311 |
|  | 5 | rs62575403 | 9 | 136493820 | 0.145423872 | 0.031107867 | -0.00746234 | 0.0560935 | 21.84947967 |
|  | 6 | rs6586324 | 21 | 43942135 | 0.047779239 | 0.010543692 | -0.000768158 | 0.0226942 | 20.53268058 |
|  | 7 | rs72706335 | 1 | 157495438 | -0.223193178 | 0.049338227 | -0.143674 | 0.0730056 | 20.45348496 |
|  | 8 | rs73512608 | 13 | 70522683 | -0.123117207 | 0.02367433 | -0.0810269 | 0.0497028 | 27.03993111 |
|  | 9 | rs73846128 | 3 | 89340254 | -0.066426181 | 0.013348134 | -0.011086 | 0.0318052 | 24.7622485 |
|  | 10 | rs73975615 | 17 | 6461200 | -0.207162752 | 0.044262873 | 0.0508691 | 0.13195 | 21.89196708 |
|  | 11 | rs7546249 | 1 | 176792690 | -0.056917331 | 0.01183824 | -0.0229617 | 0.0246572 | 23.11358942 |
|  | 12 | rs929878 | 16 | 74290641 | -0.054000099 | 0.012152588 | -0.0364091 | 0.0275219 | 19.74259275 |
