# Supplementary material for: Exploring the causal connection: insights into diabetic nephropathy and gut microbiota from whole-genome sequencing databases
Source: Ren Fail. 2024 Aug 1;46(2):2385065. doi: 10.1080/0886022X.2024.2385065 (PMC11299436; doi:10.1080/0886022X.2024.2385065)
Supplement: Supplementary Table S2.docx [file IRNF_A_2385065_SM7170.docx]

| Supplementary Table S2. MR results of causal links between the gut microbiota and DN risk (P <1 × 10-5). | | | | | | | | | | | | |  |
| --- | --- | --- | --- | --- | --- | --- | --- | --- | --- | --- | --- | --- | --- |
| Classification |  | Nsnp | Methods | Beta | SE | OR (95% CI) | P-value | Horizontal pleiotropy | | | Heterogeneity | | MR-PRESSO |
|  |  |  |  |  |  |  |  | Egger | SE | P-value | Cochran’s | P-value |  |
|  |  |  |  |  |  |  |  | Intercept |  |  | Q methods |  |  |
| class.Bacteroidia.id.912 | | 14 | MR Egger | 0.385 | 0.305 | 1.469(0.808,2.672) | 0.231 | -0.003 | 0.021 | 0.903 | MR Egger | 0.913 | 0.94 |
|  |  | 14 | Weighted median | 0.4 | 0.157 | 1.491(1.098,2.027) | 0.011 |  |  |  | IVW | 0.944 |  |
|  |  | 14 | Inverse variance weighted | 0.35 | 0.121 | 1.419(1.119,1.799) | 0.004 |  |  |  |  |  |  |
|  |  | 14 | Simple mode | 0.524 | 0.293 | 1.689(0.951,3.000) | 0.097 |  |  |  |  |  |  |
|  |  | 14 | Weighted mode | 0.536 | 0.264 | 1.709(1.019,2.865) | 0.063 |  |  |  |  |  |  |
| class.Verrucomicrobiae.id.4029 | | 12 | MR Egger | -0.123 | 0.363 | 0.884(0.435,1.800) | 0.742 | 0.04 | 0.028 | 0.184 | MR Egger | 0.501 | 0.44 |
|  |  | 12 | Weighted median | 0.377 | 0.146 | 1.457(1.094,1.942) | 0.015 |  |  |  | IVW | 0.413 |  |
|  |  | 12 | Inverse variance weighted | 0.373 | 0.106 | 1.452(1.180,1.787) | 0.004 |  |  |  |  |  |  |
|  |  | 12 | Simple mode | 0.543 | 0.259 | 1.721(1.036,2.857) | 0.053 |  |  |  |  |  |  |
|  |  | 12 | Weighted mode | 0.525 | 0.261 | 1.690(1.101,2.281) | 0.084 |  |  |  |  |  |  |
| family.Peptostreptococcaceae.id.2042 | | 15 | MR Egger | 0.177 | 0.218 | 1.194(0.779,1.830) | 0.43 | 0.002 | 0.172 | 0.901 | MR Egger | 0.746 | 0.829 |
|  |  | 15 | Weighted median | 0.239 | 0.129 | 1.271(0.987,1.636) | 0.064 |  |  |  | IVW | 0.801 |  |
|  |  | 15 | Inverse variance weighted | 0.202 | 0.094 | 1.224(1.019,1.471) | 0.031 |  |  |  |  |  |  |
|  |  | 15 | Simple mode | 0.237 | 0.188 | 1.268(0.877,1.833) | 0.227 |  |  |  |  |  |  |
|  |  | 15 | Weighted mode | 0.264 | 0.169 | 1.302(0.935,1.812) | 0.141 |  |  |  |  |  |  |
| family.Veillonellaceae.id.2172 | | 20 | MR Egger | 0.358 | 0.174 | 1.431(1.017,2.014) | 0.055 | -0.016 | 0.014 | 0.258 | MR Egger | 0.507 | 0.489 |
|  |  | 20 | Weighted median | 0.226 | 0.128 | 1.253(0.976,1.609) | 0.077 |  |  |  | IVW | 0.483 |  |
|  |  | 20 | Inverse variance weighted | 0.181 | 0.085 | 1.198(1.014,1.416) | 0.034 |  |  |  |  |  |  |
|  |  | 20 | Simple mode | 0.193 | 0.21 | 1.213(0.803,1.831) | 0.371 |  |  |  |  |  |  |
|  |  | 20 | Weighted mode | 0.267 | 0.163 | 1.306(0.950,1.796) | 0.117 |  |  |  |  |  |  |
| family.Verrucomicrobiaceae.id.4036 | | 12 | MR Egger | -0.125 | 0.363 | 0.883(0.434,1.797) | 0.738 | 0.04 | 0.278 | 0.182 | MR Egger | 0.501 | 0.45 |
|  |  | 12 | Weighted median | 0.376 | 0.154 | 1.456(1.077,1.969) | 0.015 |  |  |  | IVW | 0.412 |  |
|  |  | 12 | Inverse variance weighted | 0.373 | 0.106 | 1.452(1.180,1.787) | 0.0004 |  |  |  |  |  |  |
|  |  | 12 | Simple mode | 0.544 | 0.263 | 1.723(1.029,2.884) | 0.063 |  |  |  |  |  |  |
|  |  | 12 | Weighted mode | 0.526 | 0.265 | 1.692(1.007,2.844) | 0.073 |  |  |  |  |  |  |
| family.Victivallaceae.id.2255 | | 13 | MR Egger | -0.122 | 0.278 | 0.885(0.514,1.525) | 0.668 | -0.002 | 0.04 | 0.961 | MR Egger | 0.78 | 0.861 |
|  |  | 13 | Weighted median | -0.095 | 0.076 | 0.909(0.784,1.055) | 0.21 |  |  |  | IVW | 0.842 |  |
|  |  | 13 | Inverse variance weighted | -0.136 | 0.057 | 0.873(0.780,0.977) | 0.018 |  |  |  |  |  |  |
|  |  | 13 | Simple mode | -0.037 | 0.13 | 0.963(0.747,1.243) | 0.779 |  |  |  |  |  |  |
|  |  | 13 | Weighted mode | -0.025 | 0.136 | 0.975(0.748,1.272) | 0.856 |  |  |  |  |  |  |
| genus..Eubacteriumcoprostanoligenesgroup.id.11375 | | 13 | MR Egger | -0.914 | 0.49 | 0.401(0.153,1.049) | 0.089 | 0.042 | 0.031 | 0.2 | MR Egger | 0.41 | 0.35 |
|  |  | 13 | Weighted median | -0.272 | 0.173 | 0.762(0.543,1.068) | 0.114 |  |  |  | IVW | 0.346 |  |
|  |  | 13 | Inverse variance weighted | -0.268 | 0.132 | 0.765(0.591,0.990) | 0.042 |  |  |  |  |  |  |
|  |  | 13 | Simple mode | -0.415 | 0.305 | 0.660(0.363,1.201) | 0.199 |  |  |  |  |  |  |
|  |  | 13 | Weighted mode | -0.333 | 0.306 | 0.717(0.393,1.305) | 0.297 |  |  |  |  |  |  |
| genus.Akkermansia.id.4037 | | 12 | MR Egger | -0.123 | 0.362 | 0.884(0.435,1.799) | 0.741 | 0.016 | 0.018 | 0.39 | MR Egger | 0.982 | 0.463 |
|  |  | 12 | Weighted median | 0.376 | 0.149 | 1.457(1.088,1.951) | 0.011 |  |  |  | IVW | 0.982 |  |
|  |  | 12 | Inverse variance weighted | 0.373 | 0.106 | 1.452(1.180,1.786) | 0.0004 |  |  |  |  |  |  |
|  |  | 12 | Simple mode | 0.541 | 0.281 | 1.718(0.990,2.981) | 0.081 |  |  |  |  |  |  |
|  |  | 12 | Weighted mode | 0.519 | 0.268 | 1.680(0.993,2.842) | 0.08 |  |  |  |  |  |  |
| genus.Catenibacterium.id.2153 | | 4 | MR Egger | 1.07 | 1.245 | 2.916(0.254,33.437) | 0.48 | -0.103 | 0.16 | 0.59 | MR Egger | 0.923 | 0.913 |
|  |  | 4 | Weighted median | 0.301 | 0.121 | 1.352(1.066,1.715) | 0.013 |  |  |  | IVW | 0.902 |  |
|  |  | 4 | Inverse variance weighted | 0.271 | 0.1 | 1.312(1.079,1.594) | 0.006 |  |  |  |  |  |  |
|  |  | 4 | Simple mode | 0.31 | 0.153 | 1.364(1.010,1.841) | 0.136 |  |  |  |  |  |  |
|  |  | 4 | Weighted mode | 0.311 | 0.157 | 1.365(1.004,1.857) | 0.141 |  |  |  |  |  |  |
| genus.Clostridiumsensustricto1.id.1873 | | 8 | MR Egger | -0.088 | 0.342 | 0.915(0.468,1.791) | 0.805 | -0.018 | 0.031 | 0.581 | MR Egger | 0.568 | 0.629 |
|  |  | 8 | Weighted median | -0.324 | 0.164 | 0.723(0.525,0.997) | 0.048 |  |  |  | IVW | 0.641 |  |
|  |  | 8 | Inverse variance weighted | -0.274 | 0.125 | 0.760(0.595,0.972) | 0.029 |  |  |  |  |  |  |
|  |  | 8 | Simple mode | -0.375 | 0.254 | 0.687(0.418,1.130) | 0.183 |  |  |  |  |  |  |
|  |  | 8 | Weighted mode | -0.371 | 0.227 | 0.690(0.442,1.075) | 0.145 |  |  |  |  |  |  |
| genus.Lachnoclostridium.id.11308 | | 16 | MR Egger | 0.34 | 0.388 | 1.405(0.657,3.005) | 0.395 | -0.001 | 0.026 | 0.963 | MR Egger | 0.662 | 0.705 |
|  |  | 16 | Weighted median | 0.312 | 0.151 | 1.366(1.015,1.837) | 0.04 |  |  |  | IVW | 0.73 |  |
|  |  | 16 | Inverse variance weighted | 0.323 | 0.11 | 1.381(1.114,1.713) | 0.003 |  |  |  |  |  |  |
|  |  | 16 | Simple mode | 0.65 | 0.279 | 1.915(1.108,3.309) | 0.034 |  |  |  |  |  |  |
|  |  | 16 | Weighted mode | 0.635 | 0.273 | 1.887(1.105,3.223) | 0.034 |  |  |  |  |  |  |
| genus.Parasutterella.id.2892 | | 16 | MR Egger | 0.041 | 0.244 | 1.042(0.646,1.681) | 0.867 | 0.016 | 0.02 | 0.428 | MR Egger | 0.981 | 0.98 |
|  |  | 16 | Weighted median | 0.2 | 0.109 | 1.222(0.986,1.513) | 0.067 |  |  |  | IVW | 0.98 |  |
|  |  | 16 | Inverse variance weighted | 0.229 | 0.083 | 1.257(1.068,1.480) | 0.006 |  |  |  |  |  |  |
|  |  | 16 | Simple mode | 0.212 | 0.192 | 1.236(0.849,1.801) | 0.287 |  |  |  |  |  |  |
|  |  | 16 | Weighted mode | 0.097 | 0.169 | 1.102(0.791,1.535) | 0.575 |  |  |  |  |  |  |
| order.Bacteroidales.id.913 | | 14 | MR Egger | 0.385 | 0.305 | 1.469(0.808,2.672) | 0.231 | -0.002 | 0.021 | 0.903 | MR Egger | 0.913 | 0.935 |
|  |  | 14 | Weighted median | 0.4 | 0.163 | 1.492(1.083,2.054) | 0.014 |  |  |  | IVW | 0.944 |  |
|  |  | 14 | Inverse variance weighted | 0.35 | 0.121 | 1.419(1.119,1.799) | 0.004 |  |  |  |  |  |  |
|  |  | 14 | Simple mode | 0.524 | 0.284 | 1.689(0.969,2.945) | 0.087 |  |  |  |  |  |  |
|  |  | 14 | Weighted mode | 0.536 | 0.277 | 1.709(0.993,2.940) | 0.075 |  |  |  |  |  |  |
| order.Verrucomicrobiales.id.4030 | | 12 | MR Egger | -0.123 | 0.363 | 0.884(0.435,1.800) | 0.742 | 0.04 | 0.028 | 0.184 | MR Egger | 0.501 | 0.473 |
|  |  | 12 | Weighted median | 0.377 | 0.154 | 1.457(1.077,1.972) | 0.015 |  |  |  | IVW | 0.413 |  |
|  |  | 12 | Inverse variance weighted | 0.373 | 0.106 | 1.452(1.180,1.787) | 0.0004 |  |  |  |  |  |  |
|  |  | 12 | Simple mode | 0.543 | 0.267 | 1.721(1.020,2.901) | 0.067 |  |  |  |  |  |  |
|  |  | 12 | Weighted mode | 0.525 | 0.276 | 1.690(0.983,2.905) | 0.084 |  |  |  |  |  |  |
| Phylum.Bacteroidetes.id.905 | | 12 | MR Egger | 0.373 | 0.307 | 1.451(0.796,2.647) | 0.252 | -0.003 | 0.022 | 0.89 | MR Egger | 0.864 | 0.903 |
|  |  | 12 | Weighted median | 0.367 | 0.172 | 1.444(1.030,2.023) | 0.033 |  |  |  | IVW | 0.91 |  |
|  |  | 12 | Inverse variance weighted | 0.333 | 0.128 | 1.395(1.086,1.792) | 0.009 |  |  |  |  |  |  |
|  |  | 12 | Simple mode | 0.549 | 0.263 | 1.732(1.034,2.902) | 0.061 |  |  |  |  |  |  |
|  |  | 12 | Weighted mode | 0.557 | 0.299 | 1.746(0.972,3.316) | 0.089 |  |  |  |  |  |  |
